# Supplementary material for: Carvacrol Encapsulation in Chitosan–Carboxymethylcellulose–Alginate Nanocarriers for Postharvest Tomato Protection
Source: Int J Mol Sci. 2024 Jan 16;25(2):1104. doi: 10.3390/ijms25021104 (PMC10817085; doi:10.3390/ijms25021104)
Supplement: Supplementary file 1 [file ijms-25-01104-s001.zip › ijms-2826631-supplementary.pdf]

Carvacrol Encapsulation in Chitosan–Carboxymethylcellulose–Alginate Nanocarriers for Postharvest Tomato Protection

Eva Sánchez-Hernández [1], Alberto Santiago-Aliste [1], Adriana Correa-Guimarães [1,2], Jesús Martín-Gil [1], Rafael José Gavara-Clemente [2] and Pablo Martín-Ramos [1,*]

[1] Department of Agricultural and Forestry Engineering, ETSIIAA, Universidad de Valladolid, 34004 Pa-lencia, Spain; eva.sanchez.hernandez@uva.es (E.S.-H.); alberto.santiago@estudiantes.uva.es (A.S.-A.); adriana.correa@uva.es (A.C.-G.); jesus.martin.gil@uva.es (J.M.-G.)

[2] Packaging Group, Institute of Agrochemistry and Food Technology (IATA-CSIC), Av. Agustín Escardino, 7, 46980 Paterna, Spain; rgavara@iata.csic.es

[*] Correspondence: pmr@uva.es

SUPPLEMENTARY MATERIAL

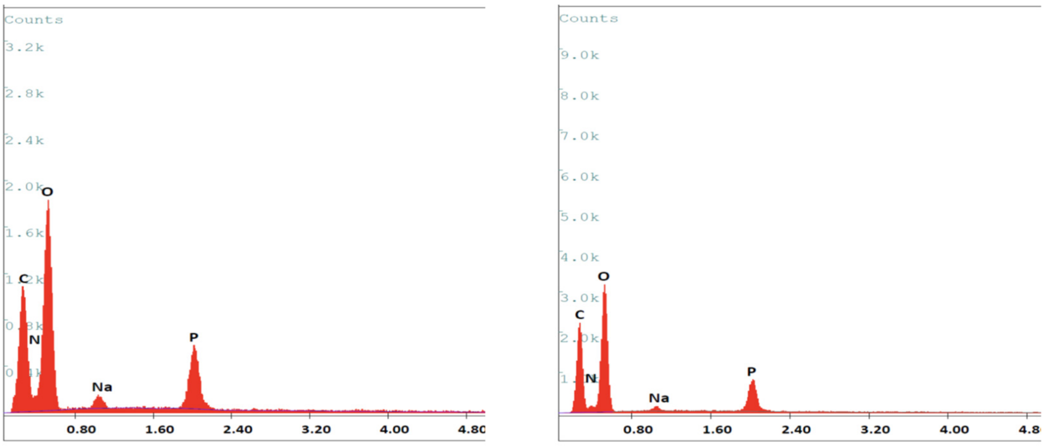

EDAX ZAF Quantification (Standardless)
Element Normalized
SEC Table : Default

Element	Wt %	At %	K-Ratio	Z
C K	34.22	43.02	0.1529	1.0262
N K	8.90	9.59	0.0283	1.0142
O K	42.39	40.01	0.1931	1.0033
NaK	1.88	1.23	0.0126	0.9293
P K	12.61	6.15	0.1116	0.9100
Total	100.00	100.00		

Element	Net Inte.	Bkgd Inte.	Inte. I
C K	138.71	1.33	1.23
N K	22.78	2.14	3.29
O K	256.05	2.92	0.91
NaK	16.75	7.26	4.81
P K	92.48	7.05	1.61

EDAX ZAF Quantification (Standardless)
Element Normalized
SEC Table : Default

Element	Wt %	At %	K-Ratio	Z
C K	37.81	47.02	0.1742	1.0248
N K	5.32	5.67	0.0161	1.0128
O K	43.58	40.69	0.2038	1.0019
NaK	1.24	0.81	0.0083	0.9279
P K	12.05	5.81	0.1066	0.9087
Total	100.00	100.00		

Element	Net Inte.	Bkgd Inte.	Inte. I
C K	152.12	0.95	0.96
N K	12.47	1.56	3.74
O K	259.91	2.48	0.74
NaK	10.67	5.78	5.22
P K	84.95	4.85	1.35

Figure S1. EDAX multi-elemental analysis results of (*left*) the empty COS–CMC–ALG NCs and (*right*) the carvacrol-loaded NCs.

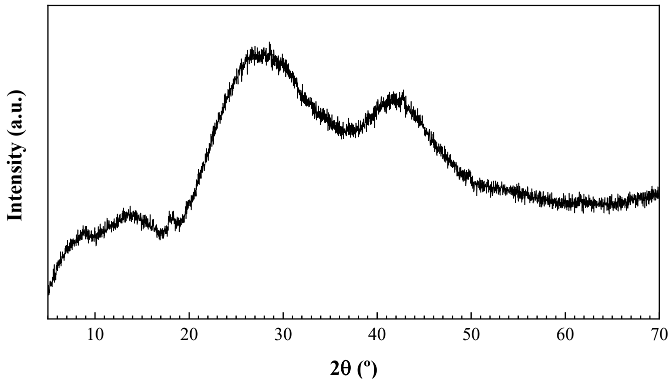

Figure S2. X-ray powder diffraction pattern of the carvacrol-loaded COS–CMC–ALG NCs.

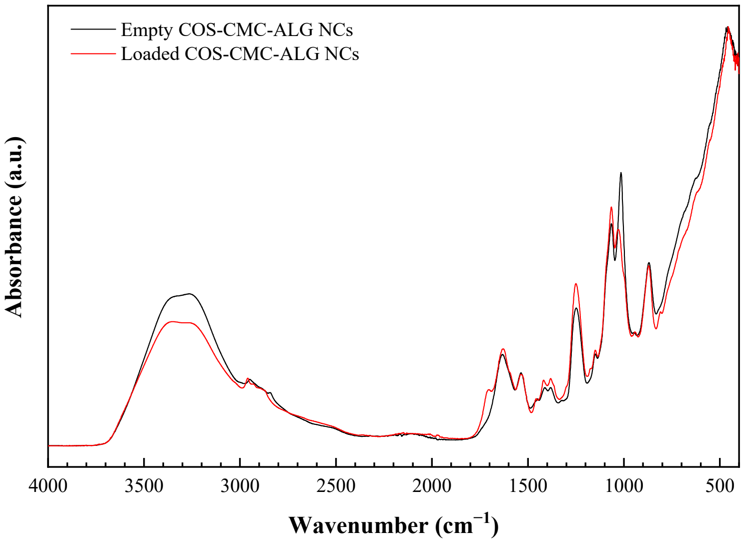

Figure S3. Infrared spectra of the empty and carvacrol-loaded COS–CMC–ALG NCs.

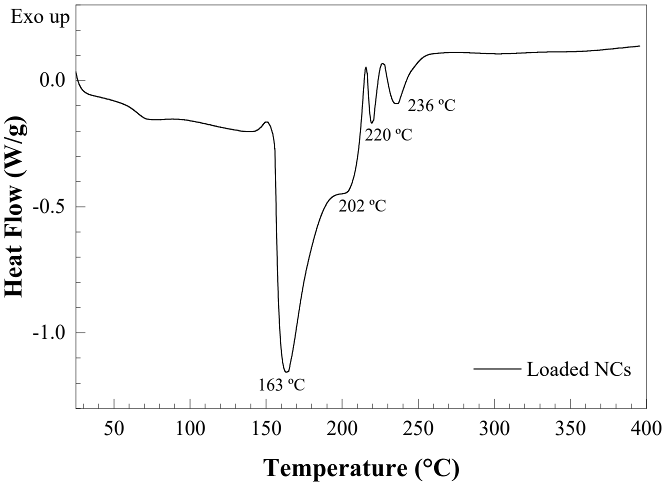

Figure S4. DSC thermograms of the carvacrol-loaded COS–CMC–ALG NCs.

Table S1. Efficacies reported in the literature for essential oils that contain carvacrol or commercial carvacrol against the three phytopathogenic fungal taxa under study.

Pathogen		Source of Carvacrol	Inhibition	Reference
B. cinerea		Commercial	MIC = 500 μg·mL^{-1}	This paper
		Commercial (98%)	MIC = 120 μL·L^{-1}	[1]
		Commercial	MIC = 100 μg·mL^{-1}	[2]
		Commercial	MIC = 300 μg·mL^{-1}	[3]
		Origanum vulgare EO (89.98% carvacrol)	EC_{50} = 9.09 μg·mL^{-1}	[4]
		O. vulgare (37.03% carvacrol)	n. a.	[5]
		O. vulgare EO (69.63-79.29% carvacrol)	MIC = 22.73 μL·L^{-1}	[6]
		Thymus serpyllum EO (54.69% carvacrol)	MIC = 22.73 μL·L^{-1}	
Colletotrichum spp.	*C. coccodes*	Commercial	MIC = 1000 μg·mL^{-1}	This paper
	C. gloeosporioides	Commercial	MIC = 1500 μg·mL^{-1}	[7]
		Commercial (95%)	MIC = 1.25 μL/paper disc	[8]
	C. fructicola	Commercial (98%)	EC_{50} = 31.97 μg·mL^{-1}	[9]
		Commercial	MIC = 400 μL L^{-1}	[10]
	C. acutatum	*Thymus vulgaris* (78.5% carvacrol)	MIC = 667 μL·L^{-1}	[11]
	-	*Lippia gracilis* EO (41.7% carvacrol)	MIC = 0.3 μL·L^{-1}	[12]
Penicillium spp.	*P. expansum*	Commercial	MIC = 1500 μg·mL^{-1}	This paper
		O. vulgare EO (66% carvacrol)	MGI = 32%, at 10 μL/disc	[13]
		Commercial	MGI = 22.2%, at 10 μL/disc	
	P. citrinum	Commercial	MIC = 150 μg·mL^{-1}	[3]
	P. chrysogenum	Commercial	MIC = 125 μg·mL^{-1}	[3]
	P. brevicompactum	*Satureja hortensis* EO (41.23% carvacrol)	IZ = 5.83 mm, at 0.75 μL·L^{-1}	[14]
		O. vulgare EO (43.26% carvacrol)	IZ = 1.67 mm, at 0.75 μL·L^{-1}	
	P. digitatum, *P. italicum*	*Origanum compactum* EO (30.5% carvacrol)	MIC = 500 μg·mL^{-1}	[15]
		Commercial	MIC = 250 μg·mL^{-1}	
	P. ochrochloron	*O. vulgare* EO (70% carvacrol)	MIC = 250 μL·L^{-1}	[16]
	P. verrucosum	*O. vulgare* subsp. *hirtum* EO (70.1% carvacrol)	MIC = 400 μg·mL^{-1}	[17]
		Commercial (99%)	MIC = 300 μg·mL^{-1}	
	P. funiculosum, *P. ochrochloron*	*Thymbra spicata* EO (74.5% carvacrol)	MIC = 0.30 μg·mL^{-1}	[18]
		Commercial	MIC = 0.25−0.125 μg·mL^{-1}	

n.a.: no activity at the highest concentration tested; EC_{50}: half maximal effective concentration; EO: essential oil; IZ: inhibition zone; MIC: minimum inhibitory concentration; MGI: mycelial growth inhibition.

References (reference numbers do not match those in the main text)

1. Zhang, J.; Ma, S.; Du, S.; Chen, S.; Sun, H. Antifungal activity of thymol and carvacrol against postharvest pathogens *Botrytis cinerea*. *Journal of Food Science and Technology* **2019**, *56*, 2611–2620, doi:10.1007/s13197-019-03747-0.
2. Tsao, R.; Zhou, T. Antifungal activity of monoterpenoids against postharvest pathogens *Botrytis cinerea* and *Monilinia fructicola*. *J. Essent. Oil Res.* **2000**, *12*, 113–121, doi:10.1080/10412905.2000.9712057.
3. Abbaszadeh, S.; Sharifzadeh, A.; Shokri, H.; Khosravi, A.R.; Abbaszadeh, A. Antifungal efficacy of thymol, carvacrol, eugenol and menthol as alternative agents to control the growth of food-relevant fungi. *Journal de Mycologie Médicale* **2014**, *24*, e51–e56, doi:10.1016/j.mycmed.2014.01.063.
4. Hou, H.; Zhang, X.; Zhao, T.; Zhou, L. Effects of *Origanum vulgare* essential oil and its two main components, carvacrol and thymol, on the plant pathogen *Botrytis cinerea*. *PeerJ* **2020**, *8*, e9626, doi:10.7717/peerj.9626.
5. Adebayo, O.; Dang, T.; Bélanger, A.; Khanizadeh, S. Antifungal studies of selected essential oils and a commercial formulation against *Botrytis cinerea*. *Journal of Food Research* **2013**, *2*, 217, doi:10.5539/jfr.v2n1p217.
6. Álvarez-García, S.; Moumni, M.; Romanazzi, G. Antifungal activity of volatile organic compounds from essential oils against the postharvest pathogens *Botrytis cinerea, Monilinia fructicola, Monilinia fructigena*, and *Monilinia laxa*. *Frontiers in Plant Science* **2023**, *14*, 1274770, doi:10.3389/fpls.2023.1274770.
7. Ochoa-Velasco, C.E.; Pérez-Pérez, J.C.; Varillas-Torres, J.M.; Navarro-Cruz, A.R.; Hernández-Carranza, P.; Munguía-Pérez, R.; Cid-Pérez, T.S.; Avila-Sosa, R. Starch edible films/coatings added with carvacrol and thymol: *In vitro* and *in vivo* evaluation against *Colletotrichum gloeosporioides*. *Foods* **2021**, *10*, 175, doi:10.3390/foods10010175.
8. Lee, D.-H.; Lee, M.-W.; Cho, S.B.; Hwang, K.; Park, I.-K. Antifungal mode of action of bay, allspice, and ajowan essential oils and their constituents against *Colletotrichum gloeosporioides* via overproduction of reactive oxygen species and downregulation of ergosterol biosynthetic genes. *Ind. Crops Prod.* **2023**, *197*, 116684, doi:10.1016/j.indcrop.2023.116684.
9. Zhao, W.; Hu, A.; Ren, M.; Wei, G.; Xu, H. First report on *Colletotrichum fructicola* causing anthracnose in chinese sorghum and its management using phytochemicals. *Journal of Fungi* **2023**, *9*, 279, doi:10.3390/jof9020279.
10. Pei, S.; Liu, R.; Gao, H.; Chen, H.; Wu, W.; Fang, X.; Han, Y. Inhibitory effect and possible mechanism of carvacrol against *Colletotrichum fructicola*. *Postharvest Biology and Technology* **2020**, *163*, 111126, doi:10.1016/j.postharvbio.2020.111126.
11. Duduk, N.; Markovic, T.; Vasic, M.; Duduk, B.; Vico, I.; Obradovic, A. Antifungal activity of three essential oils against *Colletotrichum acutatum*, the causal agent of strawberry anthracnose. *Journal of Essential Oil Bearing Plants* **2015**, *18*, 529–537, doi:10.1080/0972060x.2015.1004120.
12. Araújo, E.R.; Costa-Carvalho, R.R.; Fontes, M.G.; Laranjeira, D.; Blank, A.F.; Alves, P.B. Antifungal activity of essential oils of *Lippia* species of *Colletotrichum* sp. *in vitro*. *Acta Horticulturae* **2018**, 10.17660/ActaHortic.2018.1198.2, 9–16, doi:10.17660/ActaHortic.2018.1198.2.
13. Kadoglidou, K.; Lagopodi, A.; Karamanoli, K.; Vokou, D.; Bardas, G.A.; Menexes, G.; Constantinidou, H.-I.A. Inhibitory and stimulatory effects of essential oils and individual monoterpenoids on growth and sporulation of four soil-borne fungal isolates of *Aspergillus terreus, Fusarium oxysporum, Penicillium expansum*, and *Verticillium dahliae*. *Eur. J. Plant Pathol.* **2011**, *130*, 297–309, doi:10.1007/s10658-011-9754-x.
14. Felšöciová, S.; Vukovic, N.; Jeżowski, P.; Kačániová, M. Antifungal activity of selected volatile essential oils against *Penicillium* sp. *Open Life Sciences* **2020**, *15*, 511–521, doi:10.1515/biol-2020-0045.
15. Moussa, H.; El Omari, B.; Chefchaou, H.; Tanghort, M.; Mzabi, A.; Chami, N.; Remmal, A. Action of thymol, carvacrol and eugenol on *Penicillium* and *Geotrichum* isolates resistant to commercial fungicides and causing postharvest citrus decay. *Canadian Journal of Plant Pathology* **2020**, *43*, 26–34, doi:10.1080/07060661.2020.1767692.
16. Střelková, T.; Nemes, B.; Kovács, A.; Novotný, D.; Božik, M.; Klouček, P. Inhibition of fungal strains isolated from cereal grains via vapor phase of essential oils. *Molecules* **2021**, *26*, doi:10.3390/molecules26051313.
17. Schlösser, I.; Prange, A. Antifungal activity of selected natural preservatives against the foodborne molds *Penicillium verrucosum* and *Aspergillus westerdijkiae*. *FEMS Microbiol. Lett.* **2018**, *365*, 125, doi:10.1093/femsle/fny125.
18. Markovic, T.; Chatzopoulou, P.; Siljegovic, J.; Nikolic, M.; Glamoclija, J.; Ciric, A.; Sokovic, M. Chemical analysis and antimicrobial activities of the essential oils of *Satureja thymbra* L. and *Thymbra spicata* L. and their main components. *Archives of Biological Sciences* **2011**, *63*, 457–464, doi:10.2298/abs1102457m.
